# Multiple collapses of blastocysts after full blastocyst formation is an independent risk factor for aneuploidy — a study based on AI and manual validation

**DOI:** 10.1186/s12958-024-01242-6

**Published:** 2024-07-15

**Authors:** Lei Jin, Keyi Si, Zhou Li, Hui He, Li Wu, Bingxin Ma, Xinling Ren, Bo Huang

**Affiliations:** Reproductive Medicine Center, Tongji Hospital, Tongji Medical Colleine, Huazhong University of Science and Technology, Wuhan, 430030 People’s Republic of China

**Keywords:** Blastocyst collapse, Embryo ploidy, Time-lapse microscopy, Artificial intelligence

## Abstract

**Background:**

The occurrence of blastocyst collapse may become an indicator of preimplantation embryo quality assessment. It has been reported that collapsing blastocysts can lead to higher rates of aneuploidy and poorer clinical outcomes, but more large-scale studies are needed to explore this relationship. This study explored the characteristics of blastocyst collapse identified and quantified by artificial intelligence and explored the associations between blastocyst collapse and embryo ploidy, morphological quality, and clinical outcomes.

**Methods:**

This observational study included data from 3288 biopsied blastocysts in 1071 time-lapse preimplantation genetic testing cycles performed between January 2019 and February 2023 at a single academic fertility center. All transferred blastocysts are euploid blastocysts. The artificial intelligence recognized blastocyst collapse in time-lapse microscopy videos and then registered the collapsing times, and the start time, the recovery duration, the shrinkage percentage of each collapse. The effects of blastocyst collapse and embryo ploidy, pregnancy, live birth, miscarriage, and embryo quality were studied using available data from 1196 euploid embryos and 1300 aneuploid embryos.

**Results:**

5.6% of blastocysts collapsed at least once only before the full blastocyst formation (tB), 19.4% collapsed at least once only after tB, and 3.1% collapsed both before and after tB. Multiple collapses of blastocysts after tB (times ≥ 2) are associated with higher aneuploid rates (54.6%, *P* > 0.05; 70.5%, *P* < 0.001; 72.5%, *P* = 0.004; and 71.4%, *P* = 0.049 in blastocysts collapsed 1, 2, 3 or ≥ 4 times), which remained significant after adjustment for confounders (OR = 2.597, 95% CI 1.464–4.607, *P* = 0.001). Analysis of the aneuploid embryos showed a higher ratio of collapses and multiple collapses after tB in monosomies and embryos with subchromosomal deletion of segmental nature (*P* < 0.001). Blastocyst collapse was associated with delayed embryonic development and declined blastocyst quality. There is no significant difference in pregnancy and live birth rates between collapsing and non-collapsing blastocysts.

**Conclusions:**

Blastocyst collapse is common during blastocyst development. This study underlined that multiple blastocyst collapses after tB may be an independent risk factor for aneuploidy which should be taken into account by clinicians and embryologists when selecting blastocysts for transfer.

**Supplementary Information:**

The online version contains supplementary material available at 10.1186/s12958-024-01242-6.

## Introduction

Choosing embryos with high developmental potential is crucial for ensuring higher implantation rates (IR) and live birth rates (LBR). Since the early days of in vitro fertilization (IVF), the conventional way of morphological evaluation has been recognized generally as the mainstream non-invasive strategy for embryo evaluation by most clinicians and embryologists, which was made up of only several static microscopic observations taken at specific times during pre-implantation development [1, 2]. Time-lapse microscopy (TLM) allows embryologists to track, record and assess embryonic morphology and developmental events through real-time images, providing solutions to some of the limitations of static morphological assessment [3]. TLM can identify morphological phenomena such as irregular division and blastocyst collapse and re-expansion, which are often overlooked by static observation using conventional incubators [4, 5]. The dynamic process of embryo development can also be assessed and summarized more comprehensively with distinct morphokinetic variables [6].

TLM also enables the observation of blastocyst collapse and re-expansion. They have been seen in rabbits [7], bovines [8], mice [9], domestic cats [10] and detailed in humans by Marcos [11]. Many human blastocysts undergo one or more collapses of the blastocoel cavity, resulting in the separation of part or all of the trophectoderm (TE) cells from the zona pellucida (ZP) [11]. The occurrence of blastocyst collapse may become an indicator of preimplantation embryo quality assessment. It has been reported that collapsing blastocysts can lead to higher rates of aneuploidy and poorer clinical outcome, as well as changes in morphological quality and morphokinetic variables [12–17].

This study explores the associations between blastocyst collapse and embryo ploidy, morphological quality, morphokinetic parameters, and clinical outcomes using artificial intelligence (AI), preimplantation genetic testing (PGT), and TLM. We explored the characteristics of blastocyst collapse identified and quantified by AI in a large cohort including 3288 TLM videos of embryos that were biopsied for PGT and analyzed the associated developmental and genetic issues in the hope of helping clinicians and embryologists to select embryos.

## Materials and methods

### Study design and participants

Data from 1071 TLM-PGT cycles and 3288 biopsied blastocysts that were collected at the Reproductive Medicine Center, Huazhong University of Science and Technology Hospital from January 2019 to February 2023 were included in this study. Images of embryos were collected using the Embryoscope Plus time-lapse microscopy system (Vitrolife, Denmark) from post-insemination to biopsy and cryopreservation. All patients signed written informed consent and underwent the routine clinical treatment performed in our center. No additional intervention was performed.

### Embryo culture

All PGT cycles enrolled in this study were fertilized through intracytoplasmic sperm injection (ICSI), which has been described previously elsewhere [18]. All embryos were cultured by G1 Plus (Vitrolife, Sweden) in Embryoscope Plus time-lapse microscope system (Vitrolife, Denmark) until biopsied on day 5 or day 6. At biopsy, a laser (HAMILTON THORNE) was used to make a 5 μm hole in ZP, and 3–6 trophectoderm cells were obtained by mechanical dissection. The inner cell mass (ICM) and trophectoderm of blastocysts are graded according to the Gardner criteria [2]. The blastocyst was vitrified and warmed using Kitazato Kit.

### Time‑lapse monitoring and definitions of morphokinetic parameters

Embryoviewer software was used to analyze morphokinetic parameters of embryos cultured in the Embryoscope imaging system. A 10-minute image frequency is preset. Morphokinetic parameters were manually marked following Ciray et al. [6]: the mid-time of ICSI (t0), the appearance of two pronuclei (tPNa), time of pronuclei disappearance (tPNf), division to two to eight discrete cells (t2-t8), initiation of blastulation (tSB), full blastocyst formation time (the last frame before zona starts to thin) (tB), the second cell cycle (ECC2 = t4 – t2), the third cell cycle (ECC3 = t8 – t4), the synchronicity of the two blastomere divisions (s2 = t4 – t3) and synchronicity of the four blastomere divisions (s3 = t8 – t5).

### Next-generation sequencing and classification of ploidy

Next-generation sequencing (NGS) was used for all PGT cycles, which have been described previously [19]. For aneuploidy detection, the threshold was greater than 70%. The lower limit of euploidy was 30% for chromosomes 13, 16, 18, and 21, 50% for chromosome 19, and 40% for the other.

### The AI algorithm and measurement of the blastocyst collapse

**Fig. 1:**
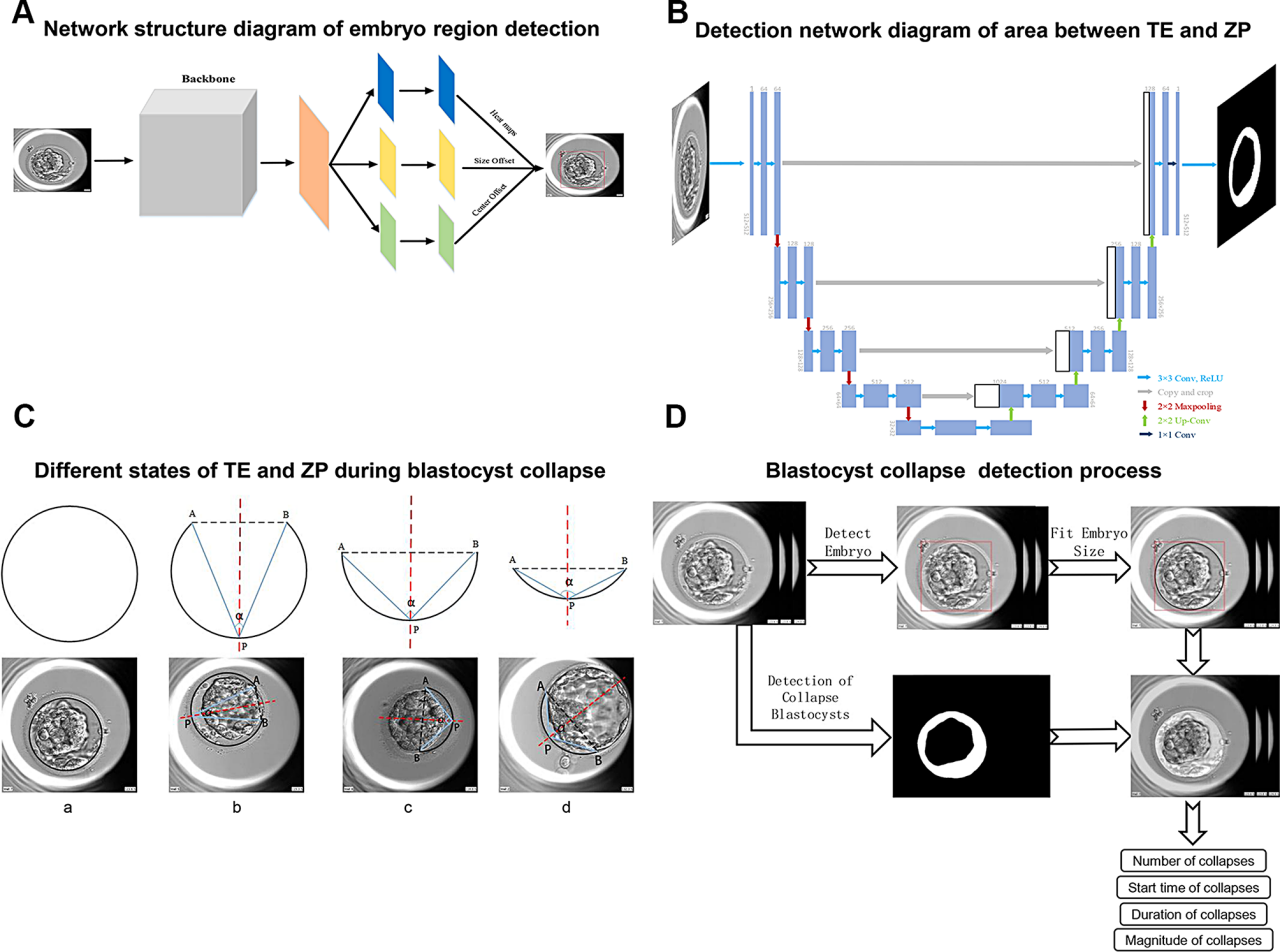
(**A**) Network structure diagram of embryo region detection: We adopt Unet [39] network to detect the blastocyst cavity area or area in zona pellucida during the blastocyst collapse. We adjust the parameters of the convolution layer in the original paper and utilize 3*3 convolution at the same level, which ensures the same size of feature maps at the same level and more efficient extraction of feature information. (**B**) Detection network diagram of area between TE and ZP:  In this artificial intelligence model, the process for detecting blastocyst collapse is as follows. Firstly, the embryo region detection network is used to calculate the embryo sequence frame by frame, predicting the period and size of the embryo. Then, the embryo at the blastocyst stage was input into the blastocyst region detection network to obtain the blastocyst cavity area or area in zona pellucida in the image, and whether the blastocyst was collapsing can be determined based on the area ratio. **(C)** Different states of TE and ZP during blastocyst collapse. **(D)** Blastocyst collapse detection process. TE, trophectoderm; ZP, zona pellucida

We build end-to-end convolutional neural networks to detect collapse (Fig. 1). Several senior embryologists divide the embryo images in the data set into the blastocyst stage and non-blastocyst stage and then mark the blastocyst area and ZP of blastocysts in the Figs. 51 and 252 pre-implantation embryo images (blastocyst stage: non-blastocyst stage = 2:1) were used to train prediction models for the blastocyst stage images and non-blastocyst stage. 24,183 blastocyst stage images (collapsing: non-collapsing = 1:3) were used to train the segmentation model of blastocyst area and zona pellucida.

**Fig. 2:**
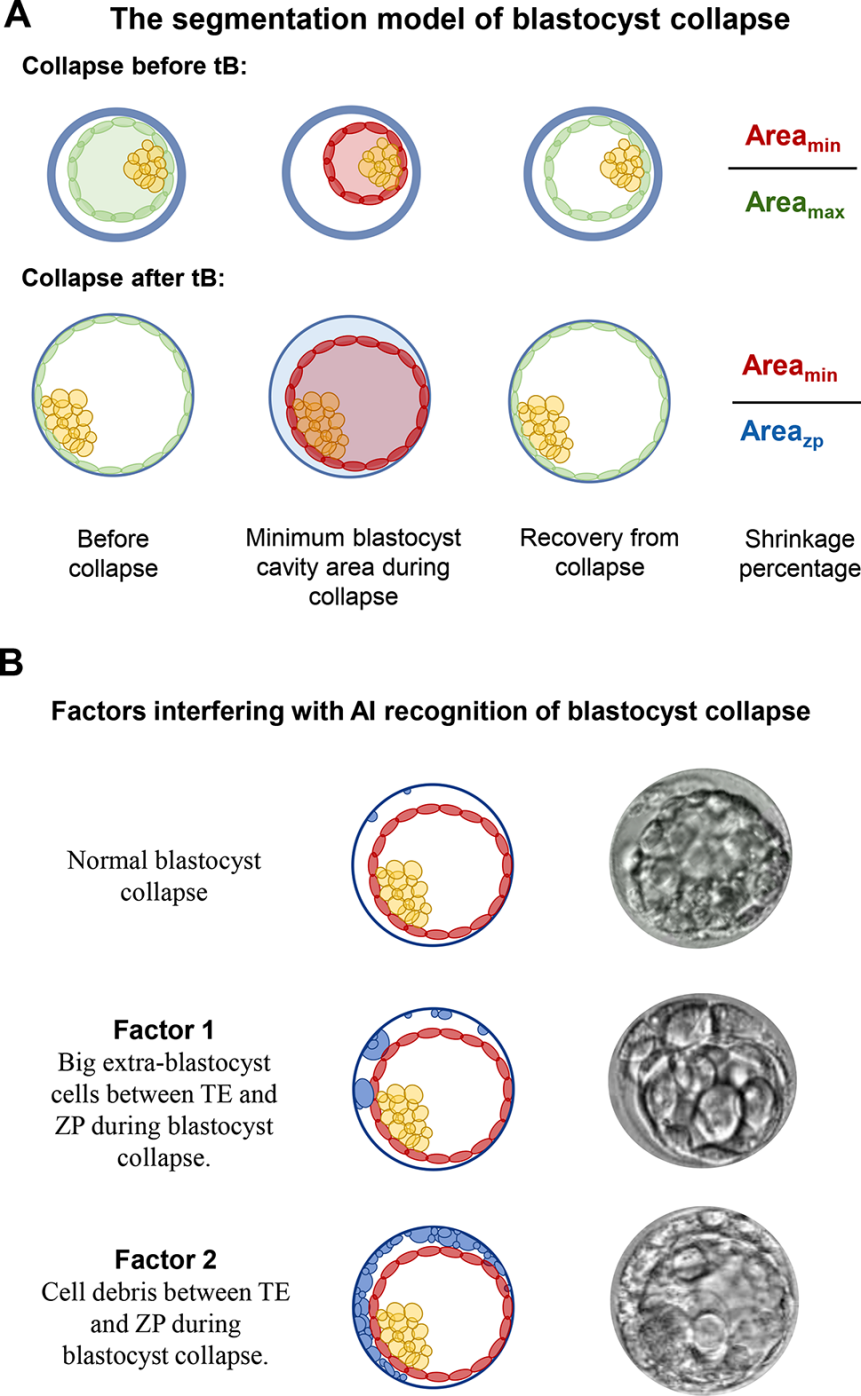
(**A**) The segmental model of blastocyst collapse. (**B**) Factors interfering with AI recognition of blastocyst collapse. ZP, zona pellucida; TE, trophectoderm

We set the successively uninterrupted blastocyst collapse as a single collapse, and determine whether a blastocyst collapsed according to the change of the area of the blastocyst (Fig. 2A). Before full blastocyst formation (tB), the ratio of minimum blastocyst cavity area to the blastocyst cavity area before a collapse event was taken as the shrinkage percentage of collapse. After tB, the ratio of minimum blastocyst cavity area to area in zona pellucida at this time was taken as the shrinkage percentage of collapse. The duration between the time of minimum blastocyst cavity area and the time of blastocyst recovery from a collapse event is recorded as the recovery duration of blastocyst collapse. The preset imaging frequency of our Embryoscope imaging system is 10 min, which limits the ability to annotate the duration of blastocyst collapse more precisely. According to the literature and practical experience [11, 13], blastocyst collapses that occur and fully recover within ten minutes are extremely rare, making this imaging frequency sufficient to identify the vast majority of blastocyst collapse events.

The results of blastocyst collapse identified by artificial intelligence were manually verified by two embryologists (Fig. 2B). Due to the 28.1% collapse rate after tSB observed in the total study sample, we selected 223 blastocysts with a collapse rate of 30%. In 10 (4.48%) blastocysts that collapsed once, the AI did not detect any collapse. In 14 (6.28%) blastocysts had multiple collapses, the AI detected fewer than the real number of collapses. The main reason for the error is that cell debris and big extra-blastocyst cells between TE and ZP affect the segmentation.

### Statistical analysis

Statistical analyses were performed using the Statistical Package for Social Sciences, version 13.0 (SPSS). Continuous variables were reported as mean ± SD and compared by Mann–Whitney U test or ANOVA. Fisher’s exact or chi-squared tests were used to compare categorical variables. The Mantel-Haenszel test was used to determine whether there was a linear trend between categorical variables.

To analyze the effects of confounders, we collected 1072 embryos with complete morphokinetic parameters and patient and cycle characteristics for multivariate analysis. Multilevel mixed-effects models account for the correlation among observations in the same cluster. Because patient-generated embryos do not provide independent information, a multi-level random effects model (level one: embryo; level two: cycle) adjusted for confounding factors such as patient and cycle characteristics was used to assess the effect of blastocyst collapse on embryo ploidy. Statistical significance was established at *P* < 0.05.

**Table 1:** Multilevel mixed-effects logistic regression model analysis for blastocyst collapse to predict the ploidy status of embryos

coefficient of constraint (*n* = 1072)	OR (95% CI)	*P* value
Numbers of blastocyst collapse after tB:		
0	Control	-
1	1.100 (0.762 to 1.588)	0.610
≥ 2	2.597 (1.464 to 4.607)	0.001
Biopsy time (day):		
5	Control	
6	1.168 (0.793 to 1.721)	0.431
ICM grade:		
A	Control	
B	1.414 (0.914 to 2.187)	0.119
TE grade:		
A	Control	-
B	1.297 (0.830 to 2.027)	0.280
C	2.567 (1.567 to 4.205)	< 0.001
t8 (hpi)	0.976 (0.939 to 1.014)	0.211
tSB (hpi)	0.955 (0.919 to 0.993)	0.020
tB (hpi)	1.063 (1.024 to 1.104)	0.002
ECC3 (h)	1.046 (0.993 to 1.102)	0.091
s3 (h)	0.949 (0.919 to 0.980)	0.001
Duration of infertility (y)	1.105 (1.035 to 1.180)	0.003
Level of AMH/ 100 (IU)	1.031 (0.989 to 1.074)	0.154

AMH, anti-Müllerian hormone; BMI, body mass index; ICM, Inner cell mass; TE, trophectoderm

### Ethical approval

All patients were given written informed consent. The study was approved by the Ethics Committee of the Reproductive Medicine Center of Tongji Hospital.

## Results

Supplemental Table S1 summarizes the main descriptive features of the cycles included in this study. The mean age of the patients was 32.0 ± 4.6 years. In 1071 TLM-PGT cycles, 1051 cycles had blastocyst formation. Supplemental Fig S1 is a flowchart depicting the process of blastocyst screening. A total of 3,288 embryos were biopsied. Among them, there were 1,381 (42.0%) euploid embryos, 1,448 (44.0%) aneuploid embryos, 416 (12.7%) mosaic embryos, and 43 (1.3%) blastocysts have no data get due to amplification failure. After removing the embryos with poor TLM image quality (57 blastocysts with imaging abnormalities such as blurring or black images and 276 blastocysts moved out of view), we analyzed the data of 1196 euploid embryos and 1300 aneuploidy embryos.

**Fig. 3:**
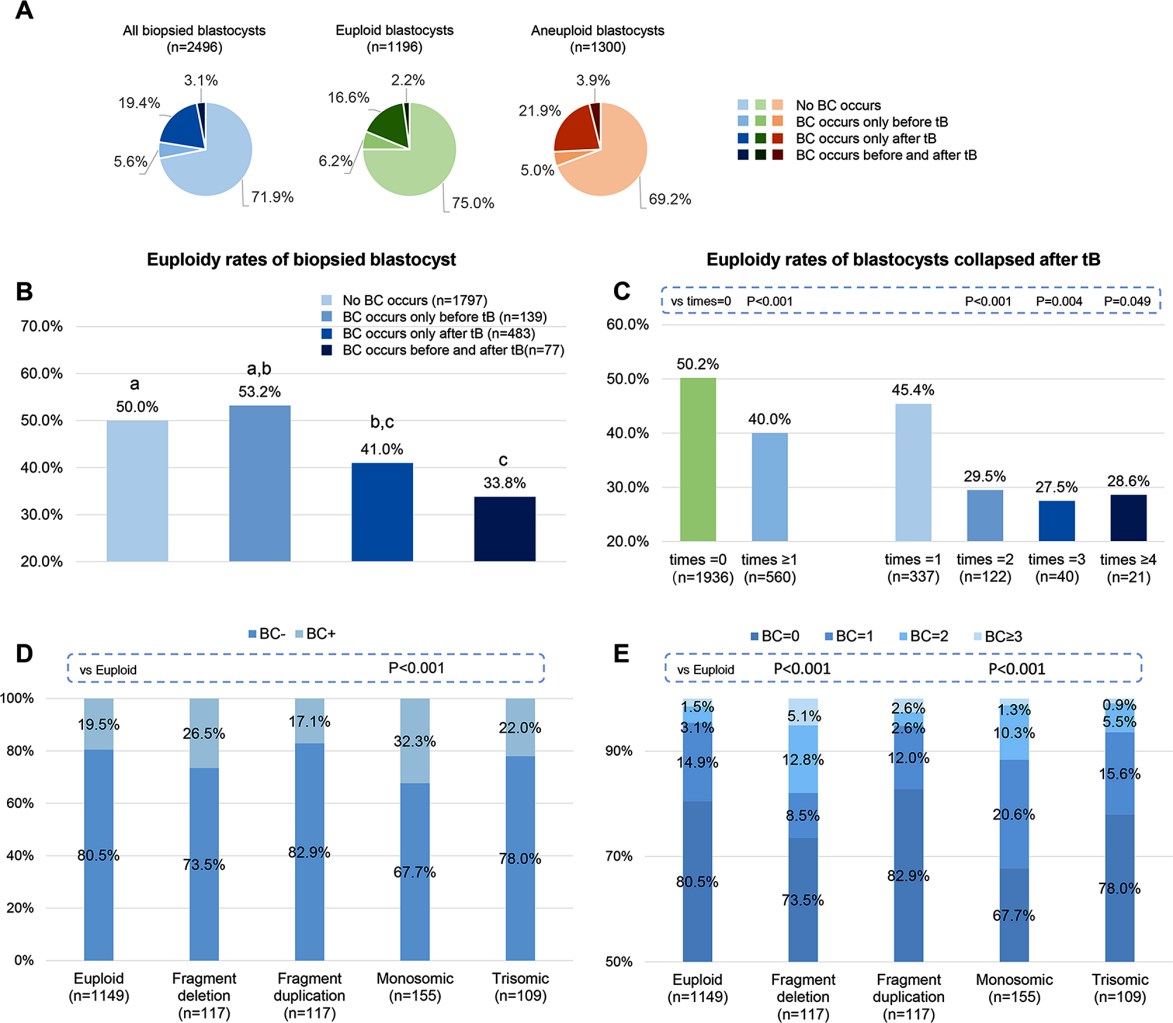
(**A**) Occurrence of blastocyst collapse in biopsied blastocysts. (**B**) Euploidy rates of biopsied blastocyst. The letters above each column display the results of pairwise comparisons between each group. (**C**) Euploidy rates of blastocysts collapsed after tB. P values for each group were obtained by comparing to embryos without blastocyst collapse. (**D, E**) Relationship between blastocyst collapse after tB and type of aneuploidy. P values for each group were obtained by comparing to euploid embryos. BC, blastocyst collapse; vs, versus

Figure 3A shows the occurrence of blastocyst collapse in all, euploid and aneuploid embryos. In this study, 5.6% of blastocysts collapsed at least once (ranging from 1 to 3) only before the time of full blastocyst formation (tB), 19.4% collapsed at least once (ranging from 1 to 6) only after tB, and 3.1% collapsed both before and after tB.

Supplementary Table S2 describes the characteristics of the first blastocyst collapse before or after tB. The first collapse before tB starts at 106.6 ± 8.2 hpi, the shrinkage percentage of which was 23.9 ± 4.5%, and the duration of which was 0.9 ± 0.9 h. For blastocysts that collapsed after tB, the first collapse starts at 121.3 ± 11.1 hpi, the shrinkage percentage of the first collapse was 25.0 ± 15.2%, and the recovery duration of the first collapse was 1.0 ± 5.8 h. There was no significant difference in the characteristics of the first blastocyst collapse between euploid and aneuploidy embryos.

### Association between blastocyst collapse and embryo ploidy

Figure 3 shows the association between blastocyst collapse and embryo ploidy. In non-collapsing blastocysts, blastocysts that only collapse before tB, blastocysts that only collapse after tB, and blastocysts that collapse both before and after tB, the euploidy rates were 50.0%, 53.2%, 41.0%, and 33.8%, respectively (Fig. 3B). We adopt the partitions of chi-squared tests to compare the euploidy rates of the four types of blastocysts at a significance level of 0.008. The euploidy rates of blastocysts that collapse after tB were significantly lower than that of non-collapsing blastocysts (*P* < 0.001 for blastocysts collapsing only after tB; *P* = 0.005 for blastocysts collapsing before and after tB). The euploidy rates of blastocysts that collapse both before and after tB were significantly lower than blastocysts that collapse only before tB (*P* = 0.006)

560 blastocysts experienced at least one collapse after tB. Their euploid rate was 40.0%, which was significantly lower than that of non-collapsing blastocysts (*P* < 0.001) (Fig. 3C). Regarding blastocysts that collapsed 1, 2, 3, or ≥ 4 times, the euploidy rates were 45.4% (*P* > 0.05), 29.5% (*P* < 0.001), 27.5% (*P* = 0.004) and 28.6% (*P* < 0.049). The euploidy rates of blastocysts that collapsed 2, 3, and more than 4 times were significantly lower than non-collapsing blastocysts. The euploidy rate of 183 blastocysts that collapsed more than once was 29.0%, significantly lower than blastocysts that collapsed only once (*P* < 0.001)

We explored whether the type of aneuploidy affected the blastocyst collapsing rate after tB (Fig. 3D). Aneuploid blastocysts are divided into 4 subgroups according to their type of chromosome variation: the fragment deletion group with subchromosomal deletion of segmental nature only, the fragment duplication group with subchromosomal duplication of segmental nature only, the monosomic group with one whole chromosome missing (there may also be subchromosomal deletion of segmental nature), and the trisomic group with one whole chromosome repeating (there may also be subchromosomal duplication of segmental nature). In comparison with euploid embryos, the monosomic group had a higher proportion of blastocyst collapse (*P* < 0.001), the fragment deletion group and the monosomic group had a higher proportion of multiple blastocyst collapses (*P* < 0.001).

### Association between blastocyst collapse and morphokinetic parameters of embryonic development

Supplementary Table S3 shows the morphokinetic parameters of non-collapsing blastocysts, blastocysts that only collapse before tB, and blastocysts that only collapse after tB. Blastocysts that only collapse before tB have delayed development in the blastocyst stages (tB, *P* < 0.001) and prolonged blastulation (tB - tSB, *P* < 0.001) compared to non-collapsing blastocysts. Blastocysts that only collapse after tB have prolonged t8 (*P* < 0.01), tSB (*P* < 0.001), tB (*P* < 0.001), ECC3 (*P* < 0.01), and s3 (*P* < 0.05) compared to non-collapsing blastocysts. For blastocysts collapse after tB, delayed t8, tSB, tB, and prolonged tB – tSB, ECC3, s3 were observed comparing subgroups with different collapsing times (1, 2, 3, or ≥ 4) and non-collapsing groups (*P* < 0.05). In particular, these morphokinetic parameters progressively extend as the collapsing times after tB rose from 0 to 2.

Since the correlation between morphokinetic parameters and embryo ploidy has been observed in previous studies [20, 21], we considered the effects of ploidy on blastocyst morphokinetic parameters in the subgroup analysis (Supplemental Table 4). In euploid embryos, we observed delayed t2 (*P* < 0.05), t4 (*P* < 0.05), tB (*P* < 0.001), and prolonged tB – tSB (*P* < 0.001) in blastocysts collapsed only before tB. In aneuploid embryos, we observed delayed tB (*P* < 0.01) and prolonged tB – tSB (*P* < 0.001) in blastocysts collapsed only before tB, and delayed t8 (*P* < 0.05), tSB (*P* < 0.01), tB (*P* < 0.001) and prolonged ECC3 (*P* < 0.05), s3 (*P* < 0.05) in blastocysts collapsed only after tB.

### Association between blastocyst collapse and embryo morphological quality

Supplementary Fig S2 shows the morphological quality of non-collapsing blastocysts, blastocysts that only collapse before tB, and blastocysts that only collapse after tB. The proportion of blastocysts biopsied on Day 5 was significantly lower in blastocysts with collapse (*P* < 0.05, Fig. 3A). The proportion of blastocysts rated as A for ICM or rated A or B for TE was significantly lower in blastocysts collapsed only after tB than in blastocysts without collapse (*P* < 0.001, Fig. 3B, 3 C). The number of blastocyst collapses after tB was negatively associated with morphological quality (*P* < 0.001 for biopsied time, ICM grade, TE grade). A similar decline of quality in blastocysts collapsed only after tB was also observed in euploid and aneuploid blastocysts (*P* < 0.01).

### Association between blastocyst collapse and clinical outcomes

**Fig. 4:**
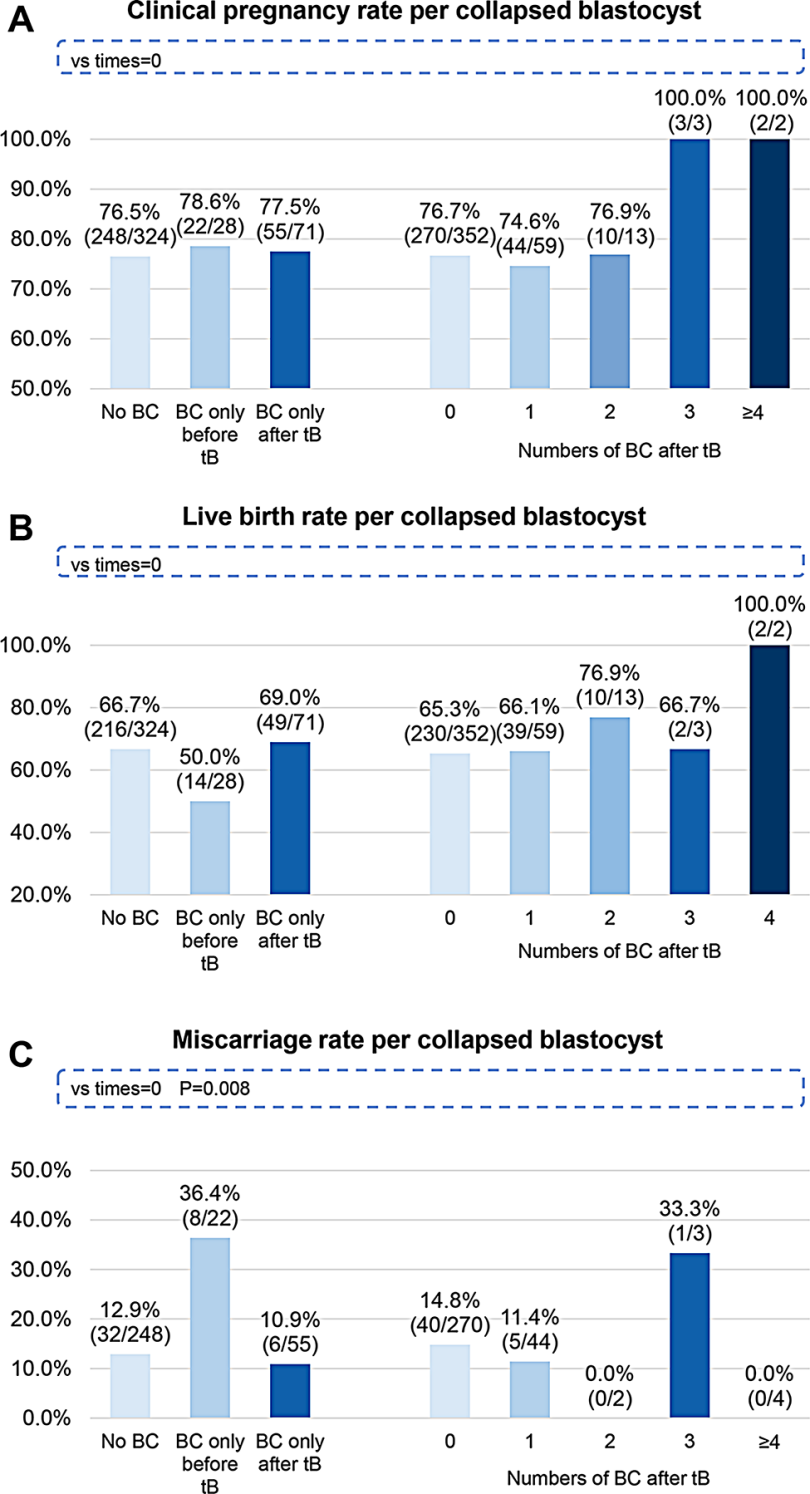
Clinical pregnancy, live birth, and miscarriage rates of 494 vitrified-warmed euploid single embryo transfers. P values for each group were obtained by comparing to embryos without blastocyst collapse. **(A)** Clinical pregnancy rate per collapsed balstocyst. **(B)** Live birth rate per collapsed blastocyst. **(C)** Miscarriage rate per collapsed blastocyst. BC, blastocyst collapse; vs, versus

Figure 4 summarizes the clinical pregnancy, live birth, and miscarriage rates of 494 vitrified-warmed euploid single embryo transfers. Whether a patient is pregnant was determined by serum β-HCG levels 9 days after the embryo transfer or the presence of fetal heart activity or gestational sac formation 7 weeks after. There is no significant difference in clinical pregnancy rates and live birth rates between collapsing and non-collapsing blastocysts (Fig. 4A and 4B). The miscarriage rate of blastocysts that collapsed only before tB was significantly higher than blastocysts without collapse (*P* = 0.008, Fig. 4C).

### Multiple collapses of blastocysts is an independent risk factor for aneuploidy

To analyze the effects of confounders on blastocyst collapse and embryo ploidy, we collected six patient and cycle characteristics (age,  body mass index (BMI), infertility duration for each female patient; level of follicle-stimulating hormone (FSH), level of anti-Müllerian hormone (AMH), and time of ovarian stimulation for each cycle) that might be associated with embryo ploidy. In the univariate analysis, six patients and cycle characteristics and morphokinetics parameters of euploid and aneuploid blastocyst were studied (Supplementary Table S5). tSB (*P* = 0.005), tB (*P* < 0.001), tSB-t8 (*P* = 0.05), tB-tSB (*P* < 0.001), infertility duration (*P* < 0.001), and level of AMH (*P* = 0.047) were significantly different between euploid and aneuploid embryos. In multilevel mixed-effects logistic regression model analysis for blastocyst collapse to predict the ploidy status (euploid or aneuploid) of embryos, morphokinetic parameters (t8, tSB, tB, ECC3, s3), time of biopsy (Day 5 or Day 6), ICM grade (A or B), TE grade (A, B or C), infertility duration, and level of AMH are taken into consideration as confounding (Table 1). Multiple blastocyst collapse (times ≥ 2) after tB remained significant (OR = 2.597, 95% CI 1.464–4.607, *P* = 0.001). In addition, TE graded C (OR = 2.567, 95% CI 1.567–4.205, *P* < 0.001), tSB (OR = 0.955, 95% CI 0.919–0.993, *P* = 0.020), tB (OR = 1.063, 95% CI 1.024–1.104, *P* = 0.002), s3 (OR = 0.949, 95% CI 0.919–0.980, *P* = 0.001), and duration of infertility (OR = 1.105, 95% CI 1.035–1.180, *P* = 0.003) also had significant effects on embryo ploidy.

## Discussion

In most studies, the definition of blastocyst collapse is a spontaneous separation of the ZP and TE in the blastocyst, resulting in the surface of the TE being separated > 50% from the inner side of the ZP [11, 17]. This definition limits the study of blastocyst collapse to blastocysts at and after the third stage (after tB). Cimadomo et al. defined collapse events as the uninterrupted reduction in the ZP area lasting < 10 h and with a final embryo: ZP ratio smaller than or equivalent to 90% and reported a high incidence of collapse events in over 50% of human embryos after tSB [13]. Using artificial intelligence, this study aimed to identify blastocyst collapse between tSB and tB, as well as blastocyst collapse after tB, and investigated whether the collapse before or after tB would have different effects on embryo ploidy and quality. In this study, 22.5% of biopsied blastocysts collapsed at least once after tB, which is similar to other studies [11, 12, 14, 16, 22]. Besides, 28.1% of biopsied blastocysts collapsed at least once after tSB, which is much smaller than Cimadomo et al. [13]. Different definitions of blastocyst collapse and different thresholds used to determine aneuploidy and euploidy in the two studies limit their comparability.

Blastocyst collapse is associated with the ploidy level of the embryo. Several studies have found lower euploidy rates of collapsing blastocysts compared with the non-collapsing blastocysts [13, 15, 16]. This study indicates that the euploid rate of blastocysts with multiple collapses after tB decreases significantly, and there is a negative correlation between the collapsing times and the euploid rate. Of note, we found no decrease in the euploid rate for blastocysts that only collapsed before tB or collapsed once after tB. Analysis of the aneuploid embryos showed a higher ratio of collapses and multiple collapses in monosomies and embryos with subchromosomal deletion of segmental nature. However, in previous studies, monosomies present fewer collapsing times [16]. More relevant research is needed to assess this association.

The occurrence of blastocyst collapse before and after tB is related to delayed blastocyst development and poor morphological quality. In blastocysts that collapsed after tB, the dynamic parameters (t8, tSB, tB, tB – tSB, ECC3, s3) and time of biopsy were significantly prolonged, and the quality of ICM and TE declined significantly. There are negative correlations between morphological quality, prolongation of dynamic parameters, and collapsing times after tB. Similarly, other studies found that as the collapsing times increase, the delay in tEB [14], tSB, and t-biopsy [13] gradually increases. Several studies have observed poorer morphological quality of collapsing embryos [12, 13]. In blastocysts that collapsed before tB, the prolongation of dynamic parameters (tB, tB – tSB) were significant, and the proportion of blastocysts biopsied on Day 5 was significantly reduced.

In our study, no decrease in euploid rate was found in blastocysts that underwent blastocyst collapse only before tB, and the decrease in embryo quality was not as significant as in blastocysts that underwent collapse only after tB. This may be because the contact between the TE and the ZP before tB is more likely to cause significant changes in surface tension of the TE layer, leading to blastocyst collapses. Therefore, euploid embryos or embryos of better quality are also more likely to collapse before tB.

Some authors have reported a decrease in implantation rate [11, 16, 22] and pregnancy rate when collapsed blastocysts were transferred in IVF cycles [22]. In a multivariate analysis, blastocyst collapse was confounded by stronger predictors and was not considered a significant predictor of LBR [14]. However, the ploidy status of the transplanted embryo in some studies is unknown [22]. In this study, collapsing blastocysts have no significant difference in clinical pregnancy and live birth rates compared with non-collapsing blastocysts in euploid single embryo transfers. The miscarriage rate of blastocysts that collapsed only before tB was significantly higher than blastocysts without collapse. Likewise, Cimadomo et al. reported that there was no significant difference in LBR and miscarriage rate between euploid collapsing and non-collapsing blastocysts [13]. Therefore, blastocyst collapse may affect clinical outcomes mainly through ploidy status, especially aneuploidy caused by the deletion of genetic material. A previous report showed that blastocyst collapse is associated with lower implantation and clinical pregnancy rates when euploid [16]. It is necessary to conduct more research to evaluate this association and to determine the impact of confounding factors on the results.

Research on the early development of mouse embryos has shown that the expansion of the blastocoel cavity is a complex process. The higher concentration of sodium ions (Na^+^) in the blastocoel cavity forms an osmotic gradient between the blastocoel cavity and the external environment which promotes extracellular fluid to enter the blastocoel cavity through the aquaporins (AQPs), leading to an increase in hydrostatic pressure in the blastocoel cavity [23]. Dumortier et al. observed that the hydrostatic pressure in the blastocyst is comparable to pressures capable of inducing hydraulic fracturing of cell-cell contacts in vitro [24, 25]. The high hydrostatic pressure promotes the establishment of paracellular sealing in the TE layer, which allows the blastocoel cavity to retain Na^+^ and water molecules that had entered the cavity, leading to the continuous accumulation of fluid inside the blastocyst [25–27]. During the process of blastocyst expansion, it is crucial to maintain a balance between hydrostatic pressure in the blastocyst cavity and the surface tension of the TE layer. If the balance is well maintained, the blastocyst will progressively expand with oscillations. Some of the contraction that occurs at this time due to alteration in epithelial permeability may be a normal process of blastocyst development. For example, with the normal insertion of dividing cells during cytokinesis, cell rounding may lead to a transient loss of paracellular sealing, resulting in focal intercellular leakage [28].

Blastocyst collapse may be an acute failure of the TE in response to gradually increasing hydrostatic pressure during progressive expansion. According to our observation, the proportion of low-quality TE (Gardner’s scheme graded C) is higher in collapsing blastocysts. The authors suggest that abnormal morphology and function of TE cells, such as abnormal paracellular sealing, abnormal contractility of TE cells, and abnormal activity of ion pumps and water channels, may cause the TE layer to be unable to withstand excessive pressure in the blastocyst cavity, which may lead to the physical transient separation of paracellular sealing. Additionally, mechanical obstacles encountered by TE during expansion, such as excluded blastomeres or cellular debris within the perivitelline space, may also lead to significant changes in hydrostatic pressure. Then, the fluid leakage in the blastocyst relaxes the tension and induces the paracellular gaps to close, allowing the blastocyst to expand again [29]. Due to the rapid leakage rate of low-viscosity liquids, the area of the blastocyst is reduced rapidly during blastocyst collapses, and most embryos gradually re-expanded within 3 h after the collapse event.

Good intercellular connection is essential to maintain the integrity of TE during blastocyst expansion. Significant frequent collapse and developmental delay were observed in mouse embryos cultured with gap junction inhibitors [30]. Inhibiting cell contractility decreases the surface tension of the blastocyst [25, 31]. The activities of ion pumps and aquaporins on the TE cell membrane, as well as the osmotic pressure in the culture medium, affect the hydrostatic pressure in the blastocoel cavity. Na^+^ / H^+^ exchangers and Na^+^ / K^+^ - ATPase play key roles in Na^+^ influx into the apical membrane and Na+- outflow from the basal membrane of trophoblast cells, respectively [23]. Inhibiting NHE3, one of the Na^+^ / H^+^ exchangers enriched in TE apical membrane, can reduce the re-expansion rate of blastocyst collapsed by cytochalasin D [32]. Adverse factors in genes, culture medium, and culture environment may affect the quality of TE through the aforementioned ways, leading to blastocyst collapse. Viñals Gonzalez et al. found that chromosomes 1, 6, and 19 showed copy gain in collapsing blastocysts, and some of the gene families involved in blastocyst formation (i.e. Na/K-ATPase pumps, adherents, and gap or tight junctions) were located on these chromosomes [16]. In our study, such differences were not significant. More research is needed to explore whether there is an abnormal expression of related genes in aneuploid blastocysts, thereby leading to blastocyst collapse. The volatile organic compounds in the culture medium and culture environment, the increase of osmotic pressure of the culture medium, and the increase of other solute concentrations in the culture medium may also have an impact on the quality of TE [33–35].

The occurrence of severe or multiple blastocyst collapses can also have adverse effects on blastocyst development, for example, embryo dehydration and energy consumption [9, 22]. Excessive tension can not only damage the cell-cell and cell-matrix adhesions but also damage the cell membrane, causing cell death and the formation of cracks in the epithelium [24, 36, 37]. Delayed blastocyst expansion associated with multiple collapses (possibly hatching) may lead to blastocyst-endometrial asynchrony, which may decrease the LBR of the fresh cycle [38].

In summary, developmental defects in the blastocyst caused by genes or other factors may make it difficult for the blastocyst to handle the gradually increasing pressure during expansion, leading to blastocyst collapse. The process of collapse and re-expansion may also cause embryo damage. The mechanism of blastocyst collapse and re-expansion is pending to be revealed. Related experiments of other mammalian embryos can provide a reference.

## Conclusions

This study used artificial intelligence to analyze TLM videos and found that the incidence of multiple blastocyst collapses after tB was an independent risk factor for aneuploidy. In addition, there was a significant association between blastocyst collapse and delayed embryonic development, and reduced morphological quality. Analysis of the aneuploid embryos showed a higher ratio of collapses and multiple collapses in monosomies and embryos with subchromosomal deletion of segmental nature. At present, we are unable to answer the causality and mechanism behind these associations. Further large sample and multicenter studies and basic studies are needed to explore the relationship between blastocyst collapse, chromosome and IVF outcomes, and the underlying mechanism. In conclusion, we suggest that blastocyst collapses should be taken into account when clinicians and embryologists select embryos for transfer.

### Electronic supplementary material

Below is the link to the electronic supplementary material.

Supplementary Material 1

Supplementary Material 2

Supplementary Material 3

Supplementary Material 4

Supplementary Material 5

Supplementary Material 6

Supplementary Material 7

## Data Availability

Due to the sensitive nature of the questions asked in this study, survey respondents were assured raw data would remain confidential and would not be shared.

## Abbreviations

AI: artificial intelligence
AMH: anti-Müllerian hormone
AQP: aquaporins
FSH: follicle-stimulating hormone
ICM: inner cell mass
ICSI: intracytoplasmic sperm injection
IR: implantation rates
IVF: in vitro fertilization
LBR: live birth rates
NGS: next-generation sequencing
PGT: preimplantation genetic testing
TE: trophectoderm
TLM: time-lapse microscopy
ZP: zona pellucida

## References

[1] Alpha Scientists in Reproductive Medicine, ESHRE Special Interest Group Embryology (2011). Istanbul consensus workshop on embryo assessment: proceedings of an expert meeting. Reprod Biomed Online.

[2] Gardner DK, Lane M, Stevens J, Schlenker T, Schoolcraft WB (2000). Blastocyst score affects implantation and pregnancy outcome: towards a single blastocyst transfer. Fertil Steril.

[3] Apter S, Ebner T, Freour T, Guns Y, Kovacic B, ESHRE Working group on Time-lapse technology (2020). Good practice recommendations for the use of time-lapse technology†. Hum Reprod Open.

[4] Jin L, Dong X, Tan W, Huang B (2022). Incidence, dynamics and recurrences of reverse cleavage in aneuploid, mosaic and euploid blastocysts, and its relationship with embryo quality. J Ovarian Res.

[5] Zaninovic N, Irani M, Meseguer M (2017). Assessment of embryo morphology and developmental dynamics by time-lapse microscopy: is there a relation to implantation and ploidy?. Fertil Steril.

[6] Ciray HN, Campbell A, Agerholm IE, Aguilar J, Chamayou S, Esbert M (2014). Proposed guidelines on the nomenclature and annotation of dynamic human embryo monitoring by a time-lapse user group. Hum Reprod.

[7] Lewis WH, Gregory PW (1929). CINEMATOGRAPHS OF LIVING DEVELOPING RABBIT-EGGS. Science.

[8] Gonzales DS, Jones JM, Pinyopummintr T, Carnevale EM, Ginther OJ, Shapiro SS (1996). Trophectoderm projections: a potential means for locomotion, attachment and implantation of bovine, equine and human blastocysts. Hum Reprod.

[9] Niimura S (2003). Time-Lapse Videomicrographic Analyses of Contractions in mouse blastocysts. J Reprod Dev.

[10] Kij B, Kochan J, Fryc K, Nizanski W, Prochowska S, Gabrys J (2020). The frequency of collapse as a predictor of feline blastocyst quality. Theriogenology.

[11] Marcos J, Perez-Albala S, Mifsud A, Molla M, Landeras J, Meseguer M (2015). Collapse of blastocysts is strongly related to lower implantation success: a time-lapse study. Hum Reprod.

[12] Sciorio R, Thong KJ, Pickering SJ (2020). Spontaneous blastocyst collapse as an embryo marker of low pregnancy outcome: a Time-Lapse study. J Bras Reprod Assist.

[13] Cimadomo D, Marconetto A, Trio S, Chiappetta V, Innocenti F, Albricci L (2022). Human blastocyst spontaneous collapse is associated with worse morphological quality and higher degeneration and aneuploidy rates: a comprehensive analysis standardized through artificial intelligence. Hum Reprod.

[14] Bodri D, Sugimoto T, Serna JY, Kawachiya S, Kato R, Matsumoto T (2016). Blastocyst collapse is not an independent predictor of reduced live birth: a time-lapse study. Fertil Steril.

[15] Gazzo E, Peña F, Valdéz F, Chung A, Velit M, Ascenzo M et al. Blastocyst contractions are strongly related with aneuploidy, lower implantation rates, and slow-cleaving embryos: a time lapse study. JBRA Assisted Reproduction [Internet]. 2019 [cited 2023 Mar 23]; https://www.jbra.com.br/trab/pub/download_trabalho.php?fileSource=/var/www/vhosts/jbra.com.br/media/trab/arq_1846&fileName=14%20-%201478%20-%20Blastocyst.pdf&id_trabalho=684 10.5935/1518-0557.20190053 PMC6993166 31524340

[16] Viñals Gonzalez X, Odia R, Cawood S, Gaunt M, Saab W, Seshadri S (2018). Contraction behaviour reduces embryo competence in high-quality euploid blastocysts. J Assist Reprod Genet.

[17] Bickendorf K, Qi F, Peirce K, Natalwala J, Chapple V, Liu Y (2023). Spontaneous collapse as a prognostic marker for human blastocysts: a systematic review and meta-analysis. Hum Reprod.

[18] Huang B, Hu D, Qian K, Ai J, Li Y, Jin L (2014). Is frozen embryo transfer cycle associated with a significantly lower incidence of ectopic pregnancy? An analysis of more than 30,000 cycles. Fertil Steril.

[19] Huang B, Tan W, Li Z, Jin L (2021). An artificial intelligence model (euploid prediction algorithm) can predict embryo ploidy status based on time-lapse data. Reprod Biol Endocrinol.

[20] Bamford T, Barrie A, Montgomery S, Dhillon-Smith R, Campbell A, Easter C (2022). Morphological and morphokinetic associations with aneuploidy: a systematic review and meta-analysis. Hum Reprod Update.

[21] Tvrdonova K, Belaskova S, Rumpikova T, Malenovska A, Rumpik D, Myslivcova Fucikova A (2021). Differences in Morphokinetic Parameters and incidence of multinucleations in Human embryos of genetically normal, abnormal and euploid embryos leading to clinical pregnancy. JCM.

[22] Sciorio R, Saura RH, Thong KJ, Algam ME, Pickering SJ, Meseguer M (2020). Blastocyst collapse as an embryo marker of low implantation potential: a time-lapse multicentre study. Zygote.

[23] Marikawa Y, Alarcon VB (2012). Creation of trophectoderm, the first epithelium, in mouse preimplantation development. Results Probl Cell Differ.

[24] Casares L, Vincent R, Zalvidea D, Campillo N, Navajas D, Arroyo M (2015). Hydraulic fracture during epithelial stretching. Nat Mater.

[25] Dumortier JG, Le Verge-Serandour M, Tortorelli AF, Mielke A, de Plater L, Turlier H (2019). Hydraulic fracturing and active coarsening position the lumen of the mouse blastocyst. Science.

[26] Watson AJ, Natale DR, Barcroft LC (2004). Molecular regulation of blastocyst formation. Anim Reprod Sci.

[27] Eckert JJ, Fleming TP (2008). Tight junction biogenesis during early development. Biochim Biophys Acta.

[28] Huang TTF, Chinn K, Kosasa T, Ahn HJ, Kessel B (2016). Morphokinetics of human blastocyst expansion in vitro. Reprod Biomed Online.

[29] Sandre O, Moreaux L, Brochard-Wyart F (1999). Dynamics of transient pores in stretched vesicles. Proc Natl Acad Sci U S A.

[30] Togashi K, Kumagai J, Sato E, Shirasawa H, Shimoda Y, Makino K (2015). Dysfunction in gap junction intercellular communication induces aberrant behavior of the inner cell mass and frequent collapses of expanded blastocysts in mouse embryos. J Assist Reprod Genet.

[31] Maître J-L, Turlier H, Illukkumbura R, Eismann B, Niwayama R, Nédélec F (2016). Asymmetric division of contractile domains couples cell positioning and fate specification. Nature.

[32] Kawagishi R, Tahara M, Sawada K, Morishige K, Sakata M, Tasaka K (2004). Na+ / H + exchanger-3 is involved in mouse blastocyst formation. J Exp Zool Comp Exp Biol.

[33] Martinez CA, Nohalez A, Ceron JJ, Rubio CP, Roca J, Cuello C (2017). Peroxidized mineral oil increases the oxidant status of culture media and inhibits in vitro porcine embryo development. Theriogenology.

[34] Wang X, Cai J, Liu L, Jiang X, Li P, Sha A (2019). Association between outdoor air pollution during in vitro culture and the outcomes of frozen-thawed embryo transfer. Hum Reprod.

[35] Swain JE (2019). Controversies in ART: considerations and risks for uninterrupted embryo culture. Reprod Biomed Online.

[36] Harris AR, Peter L, Bellis J, Baum B, Kabla AJ, Charras GT (2012). Characterizing the mechanics of cultured cell monolayers. Proc Natl Acad Sci U S A.

[37] Trepat X, Grabulosa M, Puig F, Maksym GN, Navajas D, Farré R (2004). Viscoelasticity of human alveolar epithelial cells subjected to stretch. Am J Physiol Lung Cell Mol Physiol.

[38] Franasiak JM, Forman EJ, Patounakis G, Hong KH, Werner MD, Upham KM (2018). Investigating the impact of the timing of blastulation on implantation: management of embryo-endometrial synchrony improves outcomes. Hum Reprod Open.

[39] Ronneberger O, Fischer P, Brox T, Navab N, Hornegger J, Wells WM, Frangi AF (2015). U-Net: Convolutional Networks for Biomedical Image Segmentation. Medical Image Computing and Computer-assisted intervention – MICCAI 2015.

