# Supplementary material for: Multiple collapses of blastocysts after full blastocyst formation is an independent risk factor for aneuploidy — a study based on AI and manual validation
Source: Reprod Biol Endocrinol. 2024 Jul 15;22:81. doi: 10.1186/s12958-024-01242-6 (PMC11247853; doi:10.1186/s12958-024-01242-6)
Supplement: Supplementary file 1 — Supplementary Material 1 [file 12958_2024_1242_MOESM1_ESM.docx]

**Supplementary Table S1.** Clinical characteristics of PGT cycles.

| Parameter |  |
| --- | --- |
| No. of cycles | 1,071 |
| Age (y) | 32.0 ± 4.6 |
| Duration of infertility (y) | 2.5 ± 2.2 |
| Level of FSH | 7.4 ± 2.7 |
| Level of AMH | 4.6 ± 3.4 |
| BMI | 22..0 ± 3.1 |
| Cycles with blastocysts | 1,051 |
| Time of ovarian stimulation (days) | 9.9 ± 1.8 |
| Total number of oocytes retrieved | 14,340 |
| Total number of matured oocytes | 11,470 |
| Total number of two pronucleus (2PN) | 8,835 |
| Fertilization rate (%) | 77.0 |
| Total blastocyst biopsied | 3,288 |
| Total successfully amplified blastocyst | 3,245 |
| Average number of biopsied blastocysts per cycle | 3.1 |
| No. of euploid (%) | 1,381 (42.0) |
| No. of aneuploidy (%) | 1,448 (44.0) |
| No. of mosaicism (%) | 416 (12.7) |
| No. of amplification failed (%) | 43 (1.3) |

FSH, follicle-stimulating hormone; AMH, anti-Müllerian hormone; BMI, body mass index; PN, pronucleus.
