# Supplementary material for: Multiple collapses of blastocysts after full blastocyst formation is an independent risk factor for aneuploidy — a study based on AI and manual validation
Source: Reprod Biol Endocrinol. 2024 Jul 15;22:81. doi: 10.1186/s12958-024-01242-6 (PMC11247853; doi:10.1186/s12958-024-01242-6)
Supplement: Supplementary file 2 — Supplementary Material 2 [file 12958_2024_1242_MOESM2_ESM.docx]

**Supplementary Table S2.** A comprehensive description of the first blastocyst collapses before or after tB.

|  | BC occurs before tB | | | |  | BC occurs after tB | | | |  |
| --- | --- | --- | --- | --- | --- | --- | --- | --- | --- | --- |
|  | All  (n=139) | Euploid  (n=74) | Aneuploid  (n=65) | P value |  | All  (n=560) | Euploid  (n=224) | Aneuploid  (n=336) | P value | |
| Starting time (hpi) | 106.6±8.2 | 106.6±9.1 | 106.5±7.1 | P=0.853 |  | 121.3±11.1 | 120.1±11.6 | 122.0±10.3 | P=0.075 | |
| Shrinkage percentage (%) | 23.9±4.5 | 24.0±4.5 | 23.7±4.6 | P=0.670 |  | 25.0±15.2 | 24.3±13.6 | 25.5±16.1 | P=0.723 | |
| Recovery duration (Hours) | 0.9±0.9 | 0.9±0.8 | 0.9±1.0 | P=0.250 |  | 1.0±5.8 | 1.0±5.5 | 1.1±5.9 | P=0.107 | |

Euploid blastocysts were compared with aneuploid blastocysts. BC, blastocyst collapse; hpi, hours post-insemination; tB, the time of full blastocyst formation.
