# Supplementary material for: Multiple collapses of blastocysts after full blastocyst formation is an independent risk factor for aneuploidy — a study based on AI and manual validation
Source: Reprod Biol Endocrinol. 2024 Jul 15;22:81. doi: 10.1186/s12958-024-01242-6 (PMC11247853; doi:10.1186/s12958-024-01242-6)
Supplement: Supplementary file 3 — Supplementary Material 3 [file 12958_2024_1242_MOESM3_ESM.docx]

**Supplementary Table S4.** Morphokinetic parameters of blastocyst collapse and ploidy.

| Kinetic parameter | Euploid blastocyst | | |  | Aneuploid blastocyst | | |
| --- | --- | --- | --- | --- | --- | --- | --- |
|  | No BC (n=898) | BC only before tB (n=74) | BC only after tB  (n=198) |  | No BC (n=899) | BC only before tB (n=65) | BC only after tB  (n=285) |
| tPNa (hpi) | 8.1±2.1 | 8.1±1.8 | 8.2±2.6 |  | 8.2±2.2 | 8.3±2.5 | 7.9±2.0 |
| tPNf (hpi) | 22.6±2.8 | 23.1±2.7 | 22.8±3.3 |  | 22.7±2.7 | 22.6±2.2 | 22.7±2.8 |
| t2 (hpi) | 25.2±2.8 | 25.9±3.1^*^ | 25.4±3.4 |  | 25.4±2.9 | 25.1±2.2 | 25.4±2.9 |
| t3 (hpi) | 35.5±4.3 | 35.8±4.7 | 36.8±4.8 |  | 35.7±4.0 | 36.0±3.5 | 35.9±4.2 |
| t4 (hpi) | 36.9±4.1 | 37.8±3.9^*^ | 36.9±4.7 |  | 37.1±4.1 | 37.2±4.0 | 37.1±4.4 |
| t5 (hpi) | 48.0±6.7 | 48.2±7.4 | 48.0±7.1 |  | 48.7±6.6 | 49.9±5.3 | 48.8±6.9 |
| t8 (hpi) | 55.3±8.0 | 57.6±9.3 | 55.9±8.1 |  | 55.9±8.4 | 56.4±7.7 | 57.2±9.0^*^ |
| tSB (hpi) | 97.4±7.9 | 100.0±9.5 | 98.0±8.2 |  | 98.8±8.6 | 98.6±6.0 | 100.1±8.0^**^ |
| tB (hpi) | 107.1±8.7 | 111.8±9.5^***^ | 108.2±8.8 |  | 110.0±9.5 | 112.4±7.1^**^ | 111.2±7.8^***^ |
| PN duration (h) | 14.5±2.8 | 15.9±2.5 | 14.7±2.9 |  | 14.5±2.9 | 14.2±2.8 | 14.8±2.8 |
| t2-tPNf (h) | 2.6±0.7 | 2.6±0.5 | 2.6±0.5 |  | 2.6±0.5 | 2.5±0.3 | 2.7±0.8 |
| tSB-t8 (h) | 41.8±8.3 | 42.2±8.2 | 41.9±8.3 |  | 42.9±8.8 | 42.1±7.7 | 43.0±9.8 |
| tB-tSB (h) | 9.8±3.5 | 12.1±4.6^***^ | 10.1±3.7 |  | 10.9±4.2 | 13.8±4.6^***^ | 11.1±4.3 |
| ECC2 (h) | 11.6±2.4 | 11.8±2.3 | 11.5±2.6 |  | 11.1±2.2 | 12.1±2.9 | 11.8±2.8 |
| ECC3 (h) | 18.5±6.7 | 19.8±8.2 | 19.2±6.5 |  | 18.9±6.7 | 19.3±7.2 | 20.2±7.3^*^ |
| s2 (h) | 1.3±2.6 | 1.8±3.0 | 1.2±2.4 |  | 1.3±2.3 | 1.2±2.4 | 1.2±2.1 |
| s3 (h) | 7.3±7.1 | 8.9±8.2 | 8.1±7.3 |  | 7.3±6.8 | 6.5±6.5 | 8.6±7.5^*^ |

For the group with BC only before or after tB, the subgroups were compared with the No BC group, and the groups with significant differences (P<0.05) were marked (*, P<0.05; **, P<0.01; ***, P<0.001). BC, blastocyst collapse.
