# Supplementary material for: Multiple collapses of blastocysts after full blastocyst formation is an independent risk factor for aneuploidy — a study based on AI and manual validation
Source: Reprod Biol Endocrinol. 2024 Jul 15;22:81. doi: 10.1186/s12958-024-01242-6 (PMC11247853; doi:10.1186/s12958-024-01242-6)
Supplement: Supplementary file 4 — Supplementary Material 4 [file 12958_2024_1242_MOESM4_ESM.docx]

**Supplementary Table S5.** Patient and cycle characteristics and morphokinetic parameters of the all, euploid, and aneuploid blastocyst.

|  | All  (n=1072) | Euploid (n=552) | Aneuploid  (n=520) | P value |
| --- | --- | --- | --- | --- |
| Age (y) | 31.4±3.9 | 31.2±3.6 | 31.5±4.2 | 0.626 |
| BMI (kg/m^2^) | 21.8±3.0 | 21.6±2.6 | 22.1±3.3 | 0.147 |
| Duration of infertility (y) | 2.6±2.2 | 2.3±2.0 | 2.8±2.3 | <0.001 |
| Level of FSH/ 100 (IU) | 7.3±2.7 | 7.2±2.7 | 7.3±2.6 | 0.326 |
| Level of AMH/ 100 (IU) | 6.0±3.4 | 4.8±3.3 | 5.2±3.5 | 0.047 |
| Time of ovarian stimulation (days) | 10.0±1.7 | 10.0±1.7 | 9.9±1.6 | 0.945 |
| tPNa (hpi) | 8±2.1 | 8.1±2.1 | 8±2.2 | 0.430 |
| tPNf (hpi) | 22.6±2.8 | 22.6±2.8 | 22.6±2.7 | 0.907 |
| t2 (hpi) | 25.3±2.8 | 25.2±2.9 | 25.3±2.8 | 0.699 |
| t3 (hpi) | 35.7±4 | 35.6±4.2 | 35.8±3.9 | 0.436 |
| t4 (hpi) | 37±4 | 36.9±4 | 37±4 | 0.447 |
| t5 (hpi) | 48.6±6.4 | 48.3±6.5 | 48.9±6.2 | 0.086 |
| t8 (hpi) | 56.5±8.6 | 56.4±8.7 | 56.7±8.5 | 0.260 |
| tSB (hpi) | 98±7.8 | 97.4±7.6 | 98.6±7.9 | 0.005 |
| tB (hpi) | 108.5±8.6 | 107.3±8.4 | 109.8±8.7 | <0.001 |
| PN duration (h) | 14.6±2.8 | 14.6±2.8 | 14.7±2.9 | 0.524 |
| t2-tPNf (h) | 2.6±0.5 | 2.6±0.5 | 2.6±0.4 | 0.142 |
| tSB-t8 (h) | 41.5±8.5 | 41±8.4 | 42±8.7 | 0.050 |
| tB-tSB (h) | 10.5±4 | 10±3.6 | 11.2±4.2 | <0.001 |
| ECC2 (h) | 11.7±2.2 | 11.7±2.2 | 11.7±2.3 | 0.449 |
| ECC3 (h) | 19.5±6.9 | 19.5±7.2 | 19.6±6.7 | 0.192 |
| s2 (h) | 1.3±2.4 | 1.3±2.6 | 1.3±2.3 | 0.182 |
| s3 (h) | 7.9±7.2 | 8.1±7.5 | 7.8±6.9 | 0.770 |

Euploid embryos were compared with aneuploid embryos, and the groups with significant differences (P<0.05) were marked. (*, P<0.05; **, P<0.01; ***, P<0.001). BMI, body mass index; FSH, follicle-stimulating hormone; AMH, anti-Müllerian hormone.
