# Supplementary material for: Multiple collapses of blastocysts after full blastocyst formation is an independent risk factor for aneuploidy — a study based on AI and manual validation
Source: Reprod Biol Endocrinol. 2024 Jul 15;22:81. doi: 10.1186/s12958-024-01242-6 (PMC11247853; doi:10.1186/s12958-024-01242-6)
Supplement: Supplementary file 5 — Supplementary Material 5 [file 12958_2024_1242_MOESM5_ESM.docx]

**Supplementary Table S3.** Morphokinetic parameters of the blastocyst with or without collapse.

| Kinetic parameter | All Embryo | | |  |  | Number of BC after tB | | | |
| --- | --- | --- | --- | --- | --- | --- | --- | --- | --- |
|  | No BC (n=1,797) | BC only before tB (n=139) | BC only after tB  (n=483) |  | times=0  (n=1936) | times=1  (n=337) | times=2 (n=122) | Times=3 (n=40) | times≥4 (n=21) |
| tPNa (hpi) | 8.1±2.1 | 8.2±2.1 | 8.0±2.2 |  | 8.1±2.1 | 8.0±2.4 | 8.0±2.7 | 7.9±2.4 | 8.2±1.8 |
| tPNf (hpi) | 22.6±2.8 | 22.9±2.5 | 22.7±3.0 |  | 22.7±2.7 | 22.7±3.1 | 22.9±2.8 | 22.5±2.9 | 22.8±2.7 |
| t2 (hpi) | 25.3±2.8 | 25.5±2.7 | 25.4±3.1 |  | 25.3±2.8 | 25.4±3.2 | 25.7±2.9 | 25.2±2.8 | 25.2±2.8 |
| t3 (hpi) | 35.6±4.1 | 35.9±4.2 | 35.9±4.4 |  | 35.6±4.1 | 35.9±4.5 | 35.8±4.7 | 35.9±3.4 | 35.7±3.9 |
| t4 (hpi) | 37.0±4.2 | 37.5±3.9 | 37.0±4.5 |  | 37.0±4.1 | 37.0±4.6 | 37.3±4.4 | 36.9±3.4 | 37.1±3.6 |
| t5 (hpi) | 48.3±6.7 | 49.0±6.5 | 48.5±7.0 |  | 48.4±6.6 | 48.4±7.2 | 48.4±7.3 | 48.7±6.5 | 48.7±5.7 |
| t8 (hpi) | 55.6±8.2 | 57.0±8.5 | 56.7±8.6^**^ |  | 55.7±8.2 | 56.7±8.6 | 58.2±9.2^***^ | 57.2±9.0 | 55.6±9.5 |
| tSB (hpi) | 98.6±8.3 | 99.2±8.0 | 99.3±8.1^***^ |  | 98.2±8.3 | 99.2±8.1^**^ | 100.1±8.0^**^ | 98.1±6.0 | 94.5±7.1 |
| tB (hpi) | 108.5±9.2 | 112.1±8.4^***^ | 110.0±8.4^***^ |  | 108.8±9.2 | 110.1±9.0^***^ | 111.6±7.6^***^ | 110.7±6.3^*^ | 105.9±6.3 |
| PN duration (h) | 14.5±2.8 | 14.6±2.7 | 14.8±2.8 |  | 14.5±2.8 | 14.7±2.8 | 15.0±2.8 | 14.6±3.1 | 14.6±3.4 |
| t2-tPNf (h) | 2.6±0.6 | 2.6±0.4 | 2.7±0.7 |  | 2.6±0.6 | 2.7±0.8 | 2.7±0.6^*^ | 2.7±0.5 | 2.4±0.4 |
| tSB-t8 (h) | 42.3±8.5 | 42.1±8.0 | 42.5±9.2 |  | 42.3±8.5 | 42.4±9.2 | 42.3±10.2 | 41.0±9.1 | 38.9±9.1 |
| tB-tSB (h) | 10.4±3.9 | 12.9±4.7^***^ | 10.7±4.1 |  | 10.6±4.1 | 11.0±4.5 | 11.6±4.3^*^ | 12.7±4.3^***^ | 11.4±3.1 |
| ECC2 (h) | 11.7±2.3 | 12.0±2.6 | 11.7±2.8 |  | 11.7±2.3 | 11.7±2.7 | 11.7±3.0 | 11.6±2.1 | 11.9±1.5 |
| ECC3 (h) | 18.7±6.7 | 19.6±7.7 | 19.8±7.0^**^ |  | 18.8±6.8 | 19.7±6.7^**^ | 21.1±8.0^***^ | 20.2±7.6 | 18.5±8.0 |
| s2 (h) | 1.3±2.5 | 1.5±2.7 | 1.2±2.2 |  | 1.3±2.5 | 1.2±2.2 | 1.5±2.6 | 1.1±1.4 | 1.4±2.4 |
| s3 (h) | 7.3±7.0 | 7.7±7.5 | 8.4±7.4^*^ |  | 7.3±7.0 | 8.4±7.2^*^ | 10.2±8.0^***^ | 8.2±6.9 | 6.9±7.4 |

In the group with BC, the subgroups were compared with the No BC group or the group collapsed 0 times, and the groups with significant differences (P<0.05) were marked. (*, P<0.05; **, P<0.01; ***, P<0.001). BC, blastocyst collapse.
