# Supplementary figures and images for: Multiple collapses of blastocysts after full blastocyst formation is an independent risk factor for aneuploidy — a study based on AI and manual validation
Source: Reprod Biol Endocrinol. 2024 Jul 15;22:81. doi: 10.1186/s12958-024-01242-6 (PMC11247853; doi:10.1186/s12958-024-01242-6)

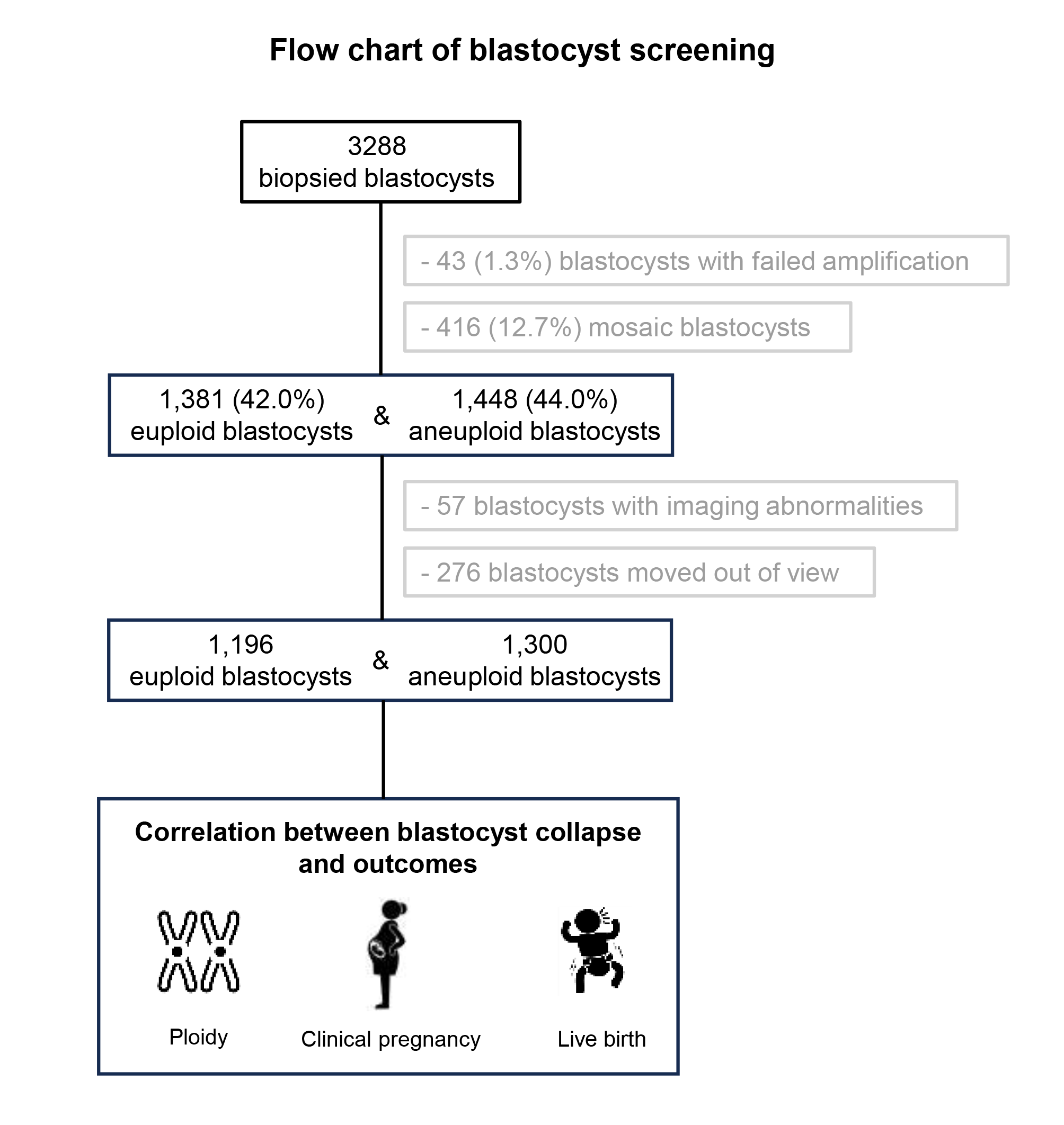

Supplement: Supplementary file 6 — Supplementary Material 6 [file 12958_2024_1242_MOESM6_ESM.tif]

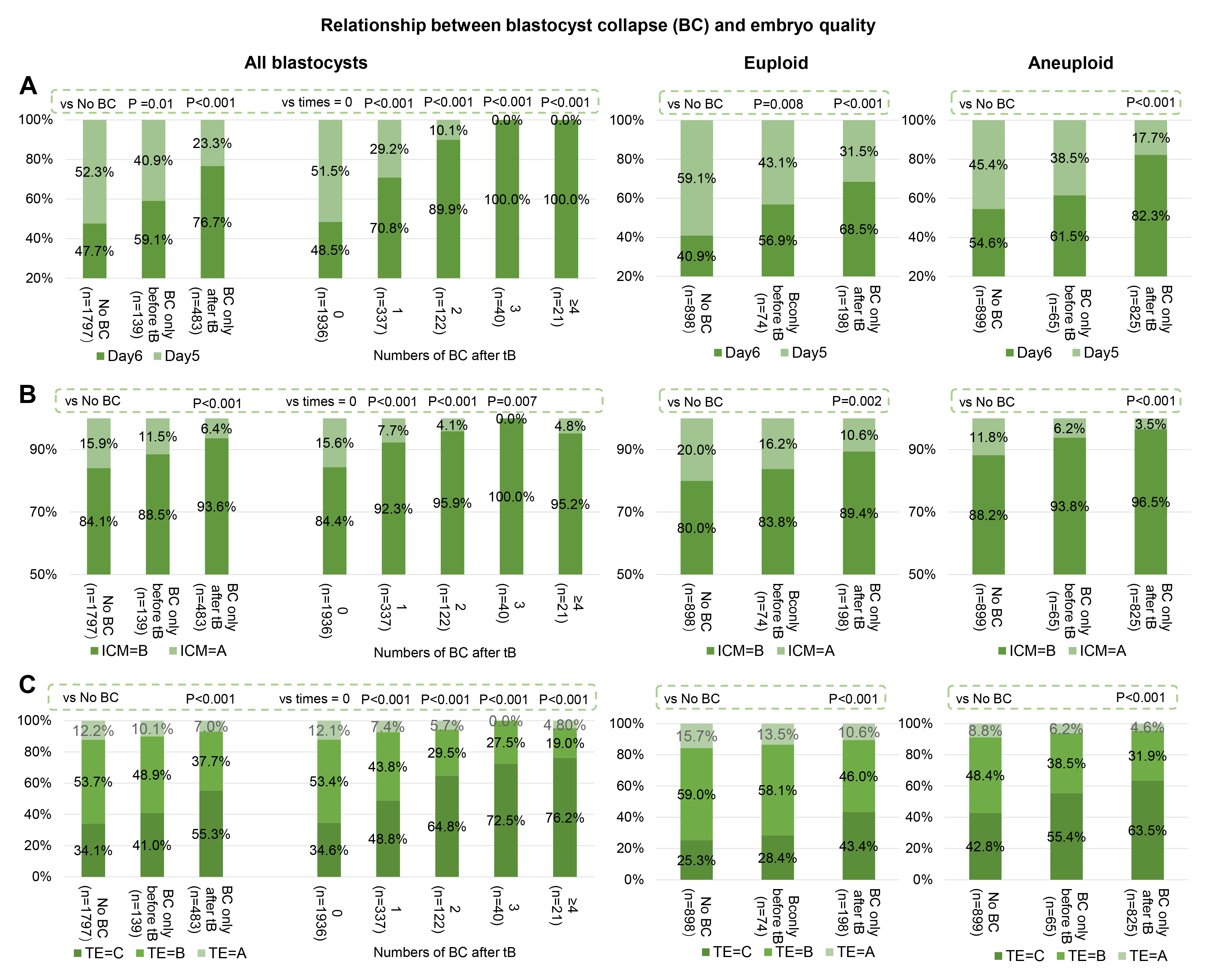

Supplement: Supplementary file 7 — Supplementary Material 7 [file 12958_2024_1242_MOESM7_ESM.tif]
